# Associative Memory Buffers the Link Between Low Life Satisfaction and High Financial Exploitation Vulnerability

**DOI:** 10.1093/geroni/igaf122.108

**Published:** 2025-12-31

**Authors:** Yaakov Hoffman, Gali Weissberger

**Affiliations:** Bar-Ilan University, Ramat Gan, HaMerkaz, Israel; Bar-Ilan University, Ramat Gan, HaMerkaz, Israel

## Abstract

Vulnerability to financial exploitation (FE) is associated with cognitive and emotional functioning. A recent MRI study demonstrated a link between the entorhinal cortex and FE vulnerability. Accordingly, the primary goal of this study was to investigate the interactive role of associative memory and life satisfaction on FE vulnerability. We focused on the false alarm memory measure; responding “yes” to a new stimulus not previously presented during a learning phase. In associative memory, the “new” stimulus is actually a re-pairing of two old stimuli that appeared during the learning phase but were not paired together. Thus a “yes” response to such stimuli (false alarm) means that participants do not distinguish well between old items and an associated pair. Older adults (N = 144, Mage 68.23±5.26, range 60-86; 63.2% female) were assessed in a cross-sectional study which included the memory test and other demographic and psychosocial variables. There was a significant main effect between lower life satisfaction and higher FE vulnerability, but no main effect of associative memory. A significant interaction showed that the link between FE vulnerability and low life satisfaction was buffered when false alarm rates for associative memory were low. Only this memory score interacted, suggesting that the ability to form and remember new associations can buffer against factors that are linked with higher financial exploitation. While these preliminary results should be further examined, they nevertheless provide an initial indication that associative learning and memory could be important protective factors against FE.

